# Ethylene glycol is metabolized to ethanol and acetate and induces expression of bacterial microcompartments in *Propionibacterium freudenreichii*

**DOI:** 10.1016/j.heliyon.2024.e33444

**Published:** 2024-06-22

**Authors:** Alexander Dank, Yue Liu, Xin Wen, Fan Lin, Anne Wiersma, Sjef Boeren, Eddy J. Smid, Richard A. Notebaart, Tjakko Abee

**Affiliations:** a Food Microbiology, Wageningen University and Research, Wageningen, Netherlands; b Laboratory of Biochemistry, Wageningen University and Research, Wageningen, Netherlands

**Keywords:** Beneficial microbe, Anaerobic, Metabolism, 1,2-Ethanediol, 1,2-Propanediol, Stress resistance

## Abstract

Ethylene glycol (EG, 1,2-ethanediol) is a two-carbon dihydroxy alcohol that can be derived from fermentation of plant-derived xylose and arabinose and which can be formed during food fermentations. Here we show that *Propionibacterium freudenreichii* DSM 20271 is able to convert EG in anaerobic conditions to ethanol and acetate in almost equimolar amounts. The metabolism of EG led to a moderate increase of biomass, indicating its metabolism is energetically favourable. A proteomic analysis revealed EG induced expression of the *pdu*-cluster, which encodes a functional bacterial microcompartment (BMC) involved in the degradation of 1,2-propanediol, with the presence of BMCs confirmed using transmission electron microscopy. Cross-examination of the proteomes of 1,2-propanediol and EG grown cells revealed PDU BMC-expressing cells have elevated levels of DNA repair proteins and cysteine biosynthesis proteins. Cells grown in 1,2-propanediol and EG also showed enhanced resistance against acid and bile salt-induced stresses compared to lactate-grown cells. Our analysis of whole genome sequences of selected genomes of BMC-encoding microorganisms able to metabolize EG with acetaldehyde as intermediate indicate a potentially broad-distributed role of the *pdu* operon in metabolism of EG. Based on our results we conclude EG is metabolized to acetate and ethanol with acetaldehyde as intermediate within BMCs in *P. freudenreichii*.

## List of abbreviations

1,2-PD: 1,2-propanediol
EG: Ethylene glycol, 1,2-ethanediol
PDU: 1,2-propandiol utilization
BMC: Bacterial microcompartment

## 1 Introduction

*Propionibacterium freudenreichii* is a generally recognized as safe (GRAS) Gram-positive bacterium which is responsible for the characteristic holes present in Swiss-type cheese by its fermentation of lactate into propionate, acetate and CO_2_ [1]. *P. freudenreichii* is used in a wide range of applications, such as *in situ* production of vitamin B_12_ in food fermentations [[2], [3], [4]] and bioreactors [5,6] and production of propionate [7]. Furthermore, *P. freudenreichii* has come into attention as potential probiotic bacterium (reviewed in Ref. [8]). The ability to utilize mucus and plant-derived 1,2-propanediol (1,2-PD) [9,10] further supports its potential as probiotic and a niche of gastro-intestinal tracts of *P. freudenreichii*.

The metabolism of 1,2-PD yields toxic intermediate propionaldehyde. Recently, evidence was provided for the wide-spread existence of proteinaceous organelles in bacteria, so called metabolosomes or bacterial microcompartments, that enable encapsulation of metabolic pathways that yield toxic intermediates [11,12], such as the propanediol utilization (*pdu*) microcompartment [13,14]. *P. freudenreichii* encodes a functional 1,2-PD utilization (*pdu*) cluster which encodes for 1,2-PD utilization by bacterial microcompartment (BMCs). The PDU BMC is induced by 1,2-PD, resulting in formation of BMCs and metabolism of 1,2-PD into propionate, ATP and 1-propanol [9].

Although the main substrate for the PDU microcompartment is 1,2-PD, its activity has been shown as well on glycerol in *Limosilactobacillus reuteri* [15]. Additionally, utilization of glycerol through a BMC-mediated pathway has been suggested for other lactic acid bacteria [16] and has been proposed to potentially occur in other *pdu-*encoding organisms as well [10]. Recently, in *Acetobacterium woodii,* metabolism of ethylene glycol (1,2-ethanediol, EG) was reported to induce expression of the *pdu* cluster [17] which results in the formation of bacterial microcompartments [18] and metabolism of EG into acetate, ATP and ethanol. PDU microcompartments may thus be implicated in metabolism of a variety of substrates. As *P. freudenreichii* also encodes a functional *pdu* cluster it is interesting to explore additional substrates able to induce these bacterial microcompartments, such as EG.

EG is a two-carbon dihydroxy alcohol that can be formed through various (native) pathways found in prokaryotes and eukaryotes that produce EG from a variety of (plant-derived) substrates such as xylose, arabinose, lyxose and serine (reviewed in Ref. [19]). EG can be formed during food fermentations, such as during bean fermentations [20,21] and vinegar production [22]. Moreover, trace amounts of EG is also consistently present in water sources, as well as human blood and urine [[23], [24], [25]]. EG can be converted in aerobic conditions through the glyoxylate pathway to glyoxylate which subsequently can be metabolized to acetyl-CoA [26]. However, also an alternative (anaerobic) pathway is described, in which a diol-dehydratase acts on EG to produce acetaldehyde [27] which subsequently is converted to either acetyl-CoA or ethanol [17]. Next, acetyl-CoA is converted to acetate by substrate-level phosphorylation yielding ATP. Hence, the ability to utilize EG under anaerobic conditions through this pathway is energetically beneficial. A drawback of this pathway however is the formation of acetaldehyde, which is toxic to bacterial cells [11] and is quickly lost from cells due to volatility [28]. As the described anaerobic catabolic EG pathway matches the BMC-mediated pathway found in *A. woodii*, we hypothesize the *pdu* cluster encodes for more broad substrate specificity in other microbes as well and hence is implicated with anaerobic metabolism of EG into acetate, ATP and ethanol.

In this study we investigated the potential of EG to be metabolized through the bacterial-microcompartment mediated *pdu*-encoded pathway in *P. freudenreichii*. In addition, a bioinformatic screening coupled to literature research pointed to broad-distributed versatility of the *pdu* cluster for a metabolic pathway that is able to convert multiple substrates. Results obtained increase our understanding of the roles of bacterial microcompartments in *P. freudenreichii* survival in specific ecosystems, such as plant-based food fermentations.

## 2 Methods

### 2.1 Strain and preculture conditions

*Propionibacterium freudenreichii* DSM 20271 was obtained from Deutsche Sammlung von Mikroorganismen und Zellkulturen (DSMZ) and routinely grown on yeast extract lactate (YEL) consisting per liter of: 10 g tryptone, 5 g yeast extract, 5 g KH_2_PO_4_ and 16 g 80 % L-Lactate syrup (Sigma Aldrich) and 15 g bacteriological agar for plates. Cell cultures were grown for 3 days in liquid media in anaerobic conditions (anoxomat modified atmosphere; 10 % CO_2_, 5 % H_2_ and 85 % N_2_) and maintained in 30 % (v/v) glycerol stocks at −80 °C. Cells were precultured for each experiment by streaking *P. freudenreichii* on YEL agar and incubating at 30 °C in anaerobic conditions for 7 days. Single colonies were inoculated in medium with composition described below.

### 2.2 Culture conditions, and growth measurement

*P. freudenreichii* DSM 20271 was grown in anaerobic jars in anaerobic conditions at 30 °C in 15 mL tubes containing 10 mL complex medium containing per liter: 10 g tryptone, 5 g yeast extract and 5 g KH_2_PO_4_, and this is referred to as the “basal medium” The basal medium was supplemented with I) no supplement, II) lactate or III) EG. All media was set at pH 7.0 by addition of 5 M NaOH and was filtered sterilized into sterile tubes through 0.2 μm sterile filters. OD_600_ measurements and extracellular metabolite samples were taken at various time intervals or only at end points (in separate experiments), for 3 independent biological replicates.

### 2.3 Analysis of organic acids

Culture samples were taken at various time intervals and were analyzed for extracellular metabolites by high performance liquid chromatography by the methods of [9]. 1 mL culture was centrifuged at 17,000×*g* for 1 min and the supernatant was collected. 0.5 mL supernatant was treated with 0.25 mL Carrez A and 0.25 mL Carrez B, vortexed and centrifuged at 17,000×*g* for 2 min. 200 μL supernatant was stored in HPLC vials at −20 °C upon analysis. 200 μL supernatant was injected on a UltiMate 3000 HPLC (Dionex Germany) equipped with an Aminex HPX-87H column (300 × 7.8 mm) with guard column (Biorad). 5 mM H_2_SO_4_ was used as mobile phase with a flow rate of 0.6 mL/min at a column temperature of 40 °C. Compounds were detected using a refractive index detector (RefractoMax 520). Quantification was performed by addition of a standard curve containing L-lactate, acetate, propionate, succinate, EG, acetaldehyde and ethanol.

### 2.4 Proteomics

Proteomic analysis was performed according to the methods of [29]. *P. freudenreichii* cells grown for 7 days on EG (50 mM) or lactate (50 mM) were harvested by centrifugation of 1 mL of sample at 17,000×*g* for 1 min in table top centrifuges and cell pellets were frozen at −80°. Samples were washed twice with 100 mM Tris (pH 8) and resuspended in 100 μl 100 mM Tris. Samples were lysed by sonication for 45 s twice while cooling 1 min on ice. Protein content was determined using Pierce Coomassie protein assay and samples were diluted to 0.5 μg/μl using Tris–HCl pH 8. Samples were prepared according to the filter assisted sample preparation protocol (FASP; [30]) with the following steps: reduction with 15 mM dithiothreitol, alkylation with 20 mM acrylamide, and digestion with sequencing grade trypsin overnight. Each prepared peptide sample was analyzed by injecting (5 μl) into a nanoLC-MS/MS (Thermo nLC1000 connected to an Orbitrap Exploris 480) as described previously [31]. LCMS data with all MS/MS spectra were analyzed with the MaxQuant quantitative proteomics software package [32] as described before [33,34] using protein database with the protein sequence of *P. freudenreichii* DSM 20271 (ID:UP000032238) downloaded from UniProt. Filtering and further bioinformatics and statistical analysis of the MaxQuant ProteinGroups file were performed with Perseus [35]. Reverse hits and contaminants were filtered out. Protein groups were filtered to contain minimally two peptides for protein identification of which at least one is unique and at least one is unmodified. Proteomics was performed on 3 independent biological replicates. Also, each group required three valid values in at least one of the two experimental groups. A volcano plot was prepared based on the Student's t-test difference between samples (FDR = 0.05). Volcano plots were produced in Rstudio using EnhancedVolcano [36]. Proteins were considered to be significantly different amongst sample if p < 0.05 and 2-fold change difference was detected.

The mass spectrometry proteomics data have been deposited to the ProteomeXchange Consortium via the PRIDE [37] partner repository with the dataset identifier PXD037369.

### 2.5 Analysis of proteomes of cells grown on 1,2-PD and EG

Data for *P. freudenreichii* grown on 1,2-PD was retrieved from PRIDE repository using identifier PXD024700. Both EG and 1,2-PD were filtered for proteins with >2 fold expression compared to lactate. Using Uniparc identifiers unique and overlapping proteins were identified.

### 2.6 Transmission electron microscopy (TEM)

*P. freudenreichii* cells grown for 7 days on EG (50 mM), or lactate (50 mM), or without supplementation, were harvested by centrifugation of 1 mL of sample at 17,000×*g* for 1 min in table top centrifuges and cell pellets were fixed by resuspending the cells in 1 mL 2.5 % (v/v) glutaraldehyde in 0.1 M sodium cacodylate buffer (pH 7.2). Further sample preparation and imaging procedures were exactly as described in Ref. [9].

### 2.7 Survival under stresses induced by low pH and bile salts

*P. freudenreichii* cells grown on media supplemented with 50 mM of EG, or 1,2-PD, or lactate, were harvested in early stationary phase by centrifugation of 1 mL culture at 2500×*g* for 5 min. The cell pellet was washed 3 times in peptone physiological salt (PPS) solution (Tritium Microbiologie, the Netherlands). For acid and bile salt treatment, the cells were resuspended in NaCl (0.5 % w/v) solution with final pH 3.3 set with HCl, or 0.2 % (w/v) bile salts (Oxoid), respectively. Cells were incubated with the stress factors at 37 °C, and at desired sampling points (specified in Results) the viability was determined by plating proper dilutions in PPS on YEL agar plates. The plates were incubated anaerobically at 30 °C for 4–5 days to determine the viable counts, with the criteria that colonies were well visible, and no additional colonies emerged with 24 h longer incubation. Control samples and stressed samples were always examined at the same time.

Statistical significance analysis was performed using one-way analysis of variance (ANOVA), and Bonferroni corrections was applied to post hoc multiple comparisons (*, p < 0.05).

## 3 Results

### 3.1 Growth performance and metabolite production on EG

**Fig. 1:**
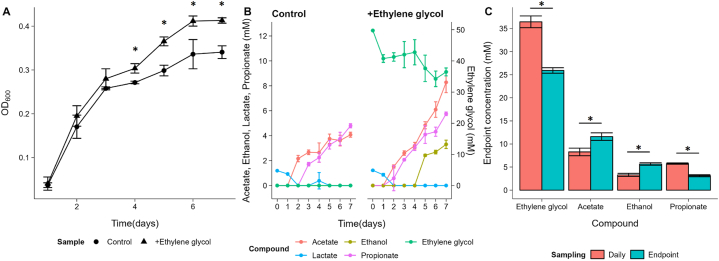
**(A)** Biomass production of *Propionibacterium freudenreichii* grown on basal medium (circles) and basal medium + 50 mM ethylene glycol (triangles). * indicates significant difference (p < 0.05) in OD between the two types of media. **(B)** Metabolite production (left y-axis) and ethylene glycol consumption (right y-axis) of *P. freudenreichii* grown on basal medium (left) and on basal medium + 50 mM ethylene glycol (right). **(C)** Substrate consumption and metabolite production at t = 7 days of *P. freudenreichii* incubated for 7 days in basal medium and basal medium supplemented with 50 mM ethylene glycol sampled either daily or only at endpoint. * indicates significance difference (p < 0.05) in metabolite concentrations between the two sampling methods. Datapoints all represent data of 3 independent biological replicates. Error bars represent standard deviation.

Anaerobic growth of P. freudenreichii on 50 mM EG was monitored for 7 days with daily sampling in triplicate. As a control, P. freudenreichii was cultured on the basal medium without additional carbon source. *P. freudenreichii* was able to grow on the basal medium without additional carbon sources, conceivably on amino acids, and on the medium supplemented with EG. Compared to the control condition a significant higher (p < 0.05, Student t-test) OD_600_ was found (Fig. 1A) for cells growing in medium supplemented with EG. In non-supplemented media an OD_600_ of 0.34 ± 0.01 was reached, whereas EG supplemented cultures reached an OD_600_ of 0.41 ± 0.01. This shows that EG supplementation results in higher biomass formation. EG can thus act as additional carbon source for *P. freudenreichii*.

Analysis of substrate consumption showed that 36.5 ± 1.3 mM EG remained after incubation for 7 days (Fig. 1B) from the initial content of 49.8 ± 0.2 mM EG, meaning 13.3 ± 1.3 mM was metabolized (26,7 % of EG). Acetate (8.3 ± 0.8 mM), succinate (2.6 ± 0.1 mM), propionate (5.7 ± 0.2 mM) and ethanol (3.3 mM ± 0.3 mM) were found in media supplemented with EG. In the non-supplemented media acetate (4.1 ± 0.2 mM), succinate (2.6 ± 0.1 mM) and propionate (4.8 ± 0.2 mM) were found, whereas ethanol was not detected. The addition of EG thus resulted in extra production of acetate (4.2 mM more detected) and ethanol (3.3 mM). Surprisingly, also a significant (p < 0.05, Student t-test) higher amount of propionate was found (Fig. 1B). Assuming that both the acetate and ethanol formed in higher amounts originate from EG metabolism, a total of 7.5 mM of EG can be accounted for. This means 6.5 mM of expected C_2_ compounds (approximately 49 %) are missing. This might be explained by loss of volatile acetaldehyde. The loss of carbon is in line with previous findings of BMC dependent 1,2-PD utilization in *P. freudenreichii* [9], where a loss of 28 % substrate (1,2-PD) derived products (propionate and propanol) is reported. The loss of C_2_ compounds seems to be associated with initial stages of growth, as in day 1 approximately 9 mM EG is missing, whereas no ethanol or additional acetate production can be observed. However, acetaldehyde was not detected (detection limit: 0.5 mM) in culture supernatants. When additional EG is taken up again after 4 days (approximately 6.3 mM), the produced ethanol (3.3 mM) and acetate (3.7 mM), with an acetate:ethanol ratio of 1.1, indicates no loss of substrate (Fig. 1B).

To correct for any potential effect of oxygen influx during sampling, we decided to perform also end point measurements to quantify substrate utilization and product formation after incubation in strictly anaerobic conditions. Analysis of samples taken after 7 days of incubation showed a significant increase in substrate utilization, i.e., 24.0 mM EG was utilized (48,4 % of EG compared to 26.7 % utilized in daily sampling). The strict anaerobic conditions resulted in production of 11.6 ± 0.8 mM acetate, 3.1 ± 0.2 mM propionate and 5.7 ± 0.3 mM ethanol (Fig. 1C). When correcting for the metabolites found in the basal medium, production of 6.9 ± 0.8 mM acetate and 5.7 ± 0.3 mM ethanol was observed, an acetate:ethanol ratio of 1.2. Similarly as in daily sampling a large proportion of expected C_2_ was missing (47.8 %). The found acetate:ethanol ratio is apparently not affected by the potential influx of oxygen while sampling daily. Again a higher concentration of propionate was found in the EG supplemented medium compared to the basal medium (Fig. 1C), supporting our earlier findings. However, the concentration of propionate was found to be much lower in strict anaerobic samples compared to samples that were sampled daily, suggesting small oxygen influx allowed utilization of additional substrates from the basal medium such as amino acids. To avoid any potential effects of oxygen on protein expression patterns strict anaerobic samples were used for analysis of the proteome.

### 3.2 Model of BMC-dependent EG metabolism in *P. freudenreichii*

**Fig. 2:**
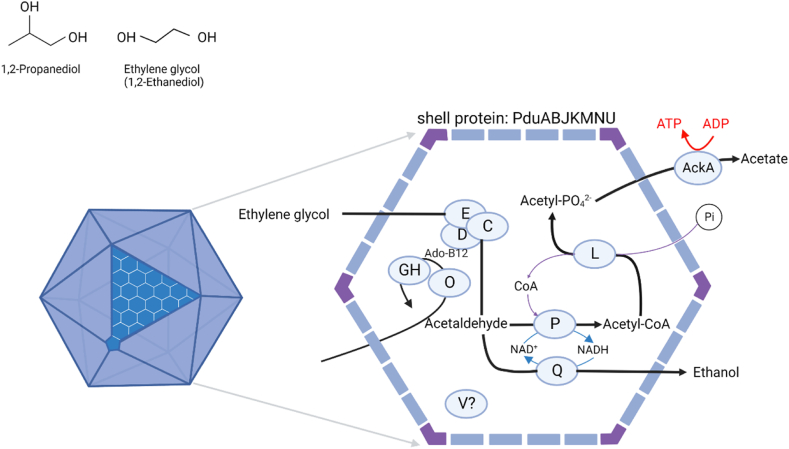
Proposed model of BMC-mediated ethylene glycol metabolism in *Propionibacterium freudenreichii*. The icosahedral diagram represents the BMC shell consisting of proteins PduABJKMNU. Inside the BMC: PduCDE, B12-dependent diol dehydratase; PduP, CoA-dependent propionaldehyde dehydrogenase; PduGH, diol dehydratase reactivase; PduO, corrinoid adenosyltransferase; PduL, phosphate propanoyltransferase; AckA, acetate kinase; PduQ, propanol dehydrogenase and PduV, of unknown function. See text for more details.

In *P. freudenreichii* two distant loci encode the *pdu* cluster. Locus 1 starts from RM25_0852 to RM25_0857 and locus 2 starts from RM25_1258 to RM25_1273 (GenBank genome accession no.CP010341). Locus 1 consists of pocR, a transcriptional regulator, *pduQ* encoding 1-propanol dehydrogenase, *pduV* with unknown function and *pduU* encoding BMC shell protein. Locus 2 consists of 14 genes including 6 BMC shell proteins (*pduABKJMN*) and 8 genes encoding enzymes for the 1,2-PD degradation pathway (*pduCDE* encodes 1,2-PD dehydratase, *pduP* encodes CoA-dependent propionaldehyde dehydrogenase, *pduL* encodes phosphate propanoyltransferase, *pduGH* (annotated as DhaG), diol dehydratase reactivase and *pduO* encodes corrinoid adenosyltransferase [9]. Notably, *pduT* a BMC-shell protein is missing from the genome of *P. freudenreichii*. PduT has been implicated in electron transport through the BMC shell [38] and was found to be non-essential for BMC assembly and functioning in *S. enterica* in laboratory conditions [39]. No PduW homologue is found in the genome of *P. freudenreichii,* instead acetate/propionate kinase (RM25_0078, AckA) is encoded on a distinct locus not associated with any other *pdu* proteins. In *A. woodii* five potential gene clusters encoding *pdu* cluster proteins involved in the formation of BMCs were identified. Several substrates were able to induce expression of the cluster encoding *pduABKNT*; 1,2-PD, 2,3-butanediol, EG and ethanol of which 1,2-PD showed the highest induction indicating this compound to be the preferred substrate [18]. Hence the *pdu*-cluster is induced during the metabolism of various substrates. Interestingly, *A. woodii* encodes multiple PduU proteins, which was hypothesized to play a role in selective entry of specific metabolites in the BMC [18]. Combined with existing knowledge on BMC formation [9,12] and on the role of the *pdu*-cluster in metabolism of various substrates in *A. woodii*, we propose a model for BMC-mediated EG metabolism in *P. freudenreichii* (Fig. 2).

Interestingly, in *P. freudenreichii* a hypothetical protein (encoded by RM25_1273) consisting of 30 amino acids is predicted adjacent to *pduA*. When using protein BLAST for this hypothetical protein against the proteome of *A. woodii* it was found to be highly similar to small parts of conserved sequences in two PduU protein encoding genes (Awo_c28920 and Awo_c26570). The role and function of this small putative protein needs to be validated, but it is plausible that it is associated with a functionality within the BMC-shell in *P. freudenreichii* (Fig. 2).

### 3.3 Proteomic analysis of EG-grown cells compared to lactate-grown cells

**Fig. 3:**
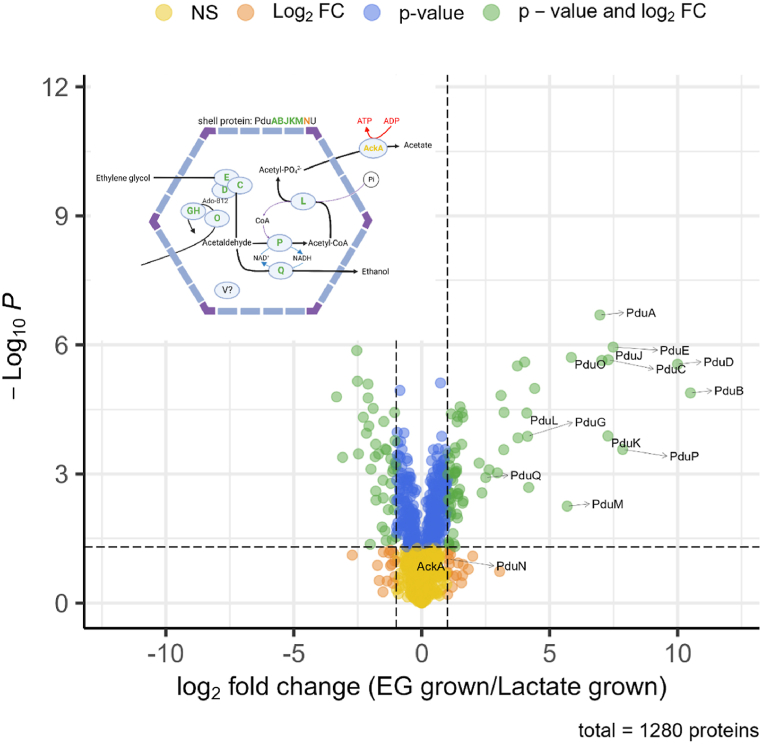
Volcano plot of proteomic analysis of cells grown in ethylene glycol media and L-lactate media. Positive log2 fold change indicate increased level in ethylene glycol-grown cells, negative log2 fold-change indicate decreased level in ethylene glycol-grown cells compared to L-lactate grown cells. Data points consist of average of three individual biological replicates. Green dots indicate proteins with p < 0.05 and 2-fold change, blue dots indicate only p < 0.05, red dots only 2-fold change in expression, orange dots indicate non-significant, non-differentially expressed proteins. Arrows indicate upregulated PDU proteins. Top left-corner; Visualization of protein expression levels in the proposed BMC-model.

A proteomic analysis was performed for cells grown on media supplemented with lactate and cells grown on media supplemented with EG (Fig. 3). Filtering of proteins with at least 2-fold increase and with a p-value <0.05 revealed 70 proteins to be more abundant in EG-grown cells compared to lactate-grown cells. Thirteen of these proteins are encoded in the *pdu* cluster and were found to be highly induced in EG-grown cells **(**Fig. 3 and Supplementary Table S1**)**. We were able to identify major BMC shell proteins PduABJKMN. We were unable to detect PduU and PduV in our proteomes. In addition to the shell proteins, we were also able to identify the complete 1,2-PD degradation pathway; 1,2-PD dehydratase (PduCDE), 1-propanol dehydrogenase (PduQ), CoA-dependent propionaldehyde dehydrogenase (PduP), phosphate propanoyltransferase (PduL) and acetate kinase (AckA). Furthermore Pdu proteins involved in B_12_ recycling were found; corrinoid adenosyltransferase (PduO) and diol dehydratase reactivase (PduGH, annotated as DhaG). Our results support the findings in *A. woodii* that EG is able to induce expression of the *pdu* cluster and is metabolized within the BMC [17,18].

**Fig. 4:**
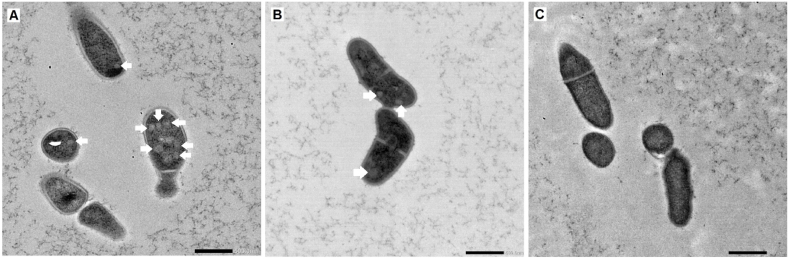
Visualization of BMCs in *P. freudenreichii* with transmission electron microscopy. **(A)** Presence of BMCs in *P. freudenreichii* cells grown in ethylene glycol media. **(B)** Presence of BMCs in *P. freudenreichii* grown in 1,2-propanediol media. **(C)** Absence of BMCs in *P. freudenreichii* grown in lactate media. Representative pictures are shown from at least 50 cells imaged per sample. White arrows indicating BMCs, all scale bars are 500 nm.

Supporting the proteomic analysis, BMC-like structures could be visualized in cells grown on media supplemented with EG (Fig. 4A), as well as in cells grown on 1, 2-PD (Fig. 4B, in line with Dank, Zeng et al. [9]), while in cells grown on lactate no BMC-like structures were present (Fig. 4C).

In line with findings of Dank, Zeng et al. [9], we found a variety of proteins with increased levels that are correlated to bacterial stress response (discussed in section below). Furthermore we found 2 proteins involved in the synthesis of pyridoxal (vitamin B_6_), PdxT and PdxS, to be strongly upregulated (21 and 16-fold). PdxT and PdxS form an enzyme complex which synthesizes pyridoxal *de novo* from glutamine together with either ribulose 5-phosphate or ribose 5-phosphate and with either glyceraldehyde-3-phosphate or dihydroxyacetone phosphate [40]. Vitamin B6 is a co-factor of many enzymatic activities including amino acid biosynthesis or catabolism. Twenty-one different enzymes binding vitamin B6 (excluding pyridoxal biosynthesis proteins) were detected in the proteome of *P. freudenreichii* grown on EG, of which cysteine synthase (encoded by RM25_1561) was found to be significantly more abundant (8-fold) compared to cells growing on lactate. In addition to cysteine synthase, glutamate decarboxylase GadD and branched-chain aminotransferase IlvE were the only proteins that had both >2 fold abundance and p < 0.05 to require vitamin B6 as co-factor. The increased levels of pyridoxal producing proteins thus may result from a higher requirement of pyridoxal (derivatives). Beside cysteine synthase, two other sulphur-metabolism related proteins also showed increased levels: sulfite:ferredoxin reductase (encoded by RM25_2032, 9-fold) and sulphate adenylyltransferase (13-fold) (discussed below).

When examining proteins down-regulated in EG grown cells as compared to lactate, we found most of them are related to bacterial growth speed or bacterial growth mode including RNAses, DNAses, Wood-Werkman cycle, and electron transfer chains including ATPase, in line with the shift from lactate metabolism to EG metabolism and the corresponding slower growth compared to lactate.

### 3.4 Identification of proteins with increased abundance in both 1,2-PD and EG grown cells

**Fig. 5:**
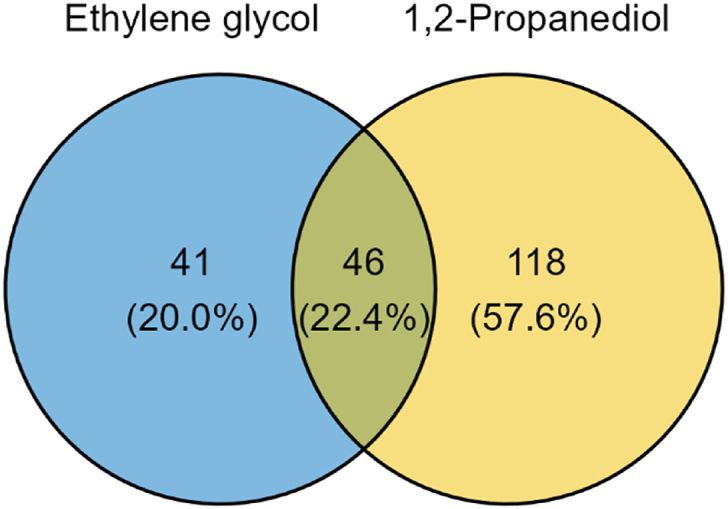
Venn diagram of proteins upregulated in ethylene glycol grown cells or 1,2-propanediol grown cells compared to L-lactate grown cells. Overlapping circles indicate proteins upregulated in both ethylene glycol as 1,2-propanediol grown cells compared to L-lactate grown cells. For a list of proteins see Supplementary Table S2.

The switch from a metabolic mode revolving around lactate towards a BMC-mediated metabolism is likely to result in expression of additional proteins besides the *pdu* cluster. Since both 1,2-PD and EG induce expression of the *pdu*-cluster and visible BMC formation (Fig. 4A and B)these proteins should be upregulated in proteome samples of both cells compared to lactate-grown cells. To identify the proteins which are strongly associated with expression of the BMCs, we combined the proteomic data for both 1,2-PD (from Dank, Zeng et al. [9] and EG grown cells and filtered these datasets for proteins with less stringent criteria (at least a 2-fold increase compared to lactate-grown cells). For 1,2-PD cells this results in 164 proteins with > 2-fold increase compared to lactate, for EG-grown cells in 87 proteins with > 2-fold increase compared to lactate. Of these proteins, 46 proteins increased more than 2-fold in both 1,2-PD-grown and EG-grown cells (Fig. 5 and Supplementary Table S2), with 13 of these proteins encoded within the *pdu* cluster (PduABCDEGJKLMOPQ).

Amongst the remaining shared proteins of 1,2-PD and EG-grown cells, multiple proteins with increased levels have putative functions in DNA and RNA synthesis and repair processes. These proteins include: a UvrD/REP helicase (encoded by RM25_1190) and ATP-dependent DNA helicase (RM25_1191); UvrD belongs to the UvrABC system responsible for nucleotide excision repair, and UvrABC system protein B (RM25_1449) also showed increased abundance. UvrD is a protein possessing ATP-dependent DNA unwinding activities and important for repair, recombination, and replication of DNA [41]. Moreover, a formamidopyrimidine-DNA glycosylase (RM25_0677) that functions as base excision repair ligase [42] and RadA (RM25_1921), a protein involved in genomic recombination and DNA repair [43,44] were also more abundant in *pdu*-induced cells. In addition to several DNA repair proteins, also RtcB (RM25_2257) was found to be more abundant. RtcB is a RNA ligase expressed to mitigate stress-induced RNA damage [45]. Our results suggest the metabolism of 1,2-PD and EG through a BMC-mediated pathway requires additional synthesis of DNA and RNA repair proteins and thus agrees with previous findings [9,14,46].

Just like in EG-grown cells, also 1,2-PD-grown cells showed increased levels of PdxT and PdxS. It thus seems pyridoxal plays an important role during metabolism of BMC-inducing substrates in *P. freudenreichii*. Cells growing on either 1,2-PD and EG also produced more AldA (encoded by RM25_1838), a putative aldehyde dehydrogenase. Assuming this protein is not associated with the BMC in *P. freudenreichii* this would imply cells growing on BMC substrates also activate alternative options to ‘capture’ aldehydes formed outside of the BMC by promiscuous enzymes or formed by incomplete BMCs. Strong induction of various sulphur-related metabolic reactions is also observed in cells growing in 1,2-PD and EG. A cysteine synthase (RM25_1561) is highly upregulated, together with a sulphite:ferredoxin reductase (RM25_2032) and a sulphate adenylyltransferase (RM25_2034). This points towards upregulation of the assimilatory sulphate reduction pathway used to convert inorganic sulphate to sulfide, which is further incorporated into carbon skeletons of amino acids as cysteine or homocysteine [47,48]. This is further supported by the increase of a methionine transporter (RM25_0298), which indicates either higher demand for methionine directly or indirectly in the form of sulphur-containing amino acid. In addition, the increase of DsbA oxidoreductase (RM25_0775), a protein responsible for disulfide-bond formation and hence correct protein folding [49], points towards additional cell requirements in either *de novo* protein formation or salvaging of damaged proteins in BMC-induced cells.

Combining these results it can be concluded the BMC-mediated metabolism is associated with additional levels of stress, resulting in a necessity to upregulate DNA, RNA and protein repair processes and consequently pathways providing the building blocks for these processes.

### 3.5 Survival of lactate, 1,2-PD and EG grown cells under stresses

**Fig. 6:**
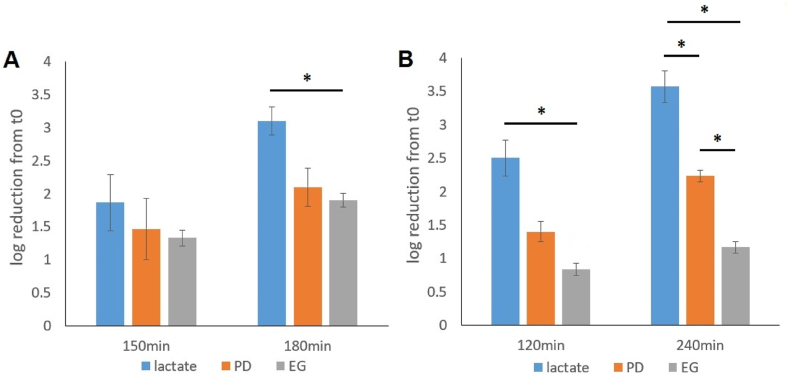
Reduction of viability in *P. freudenreichii* cultivated in lactate, 1,2-propanediol (PD) and ethylene glycol (EG) media, under **(A)** acid stress (pH 3.3) and **(B)** Bile salt (0.2 %) exposure. Log reduction values shown are calculated from at least 3 biological replicates, error bars show standard deviations of the mean (*, p < 0.05).

Following the proteomic analysis that revealed increased levels of stress response proteins in both 1,2-PD and EG-grown cells as compared to lactate-grown cells, *P. freudenreichii* cells from the respective cell cultures were exposed to selected stress conditions and the reduction in cell viability was determined (Fig. 6).

When exposed to stresses induced by acid at pH 3.3 (Figs. 6A) and 0.2 % bile salts (Fig. 6B), cells grown on EG and 1,2-PD both showed in general lower log reductions as compared to that on lactate throughout the exposure, implying higher stress resistance. This observation is in line with the increased abundance of stress response proteins in both 1,2-PD and EG-grown cells as revealed by proteomic analysis. When facing bile salt-induced stress, EG-grown cells even showed higher resistance than 1,2-PD-grown cells (Fig. 6B), which could be explained by proteins that are uniquely increased in EG-grown cells, including the ones involved in transcriptional regulation (encoded by RM25_0690, RM25_0907) and DNA repair (RM25_1022). All in all, the results showed clear effects of the stress proteins with increased abundance, in (cross)protection against acid and bile salt-induced stresses in 1,2-PD and EG-grown cells.

## 4 Discussion

*Propionibacterium freudenreichii* encodes a functional *pdu* cluster that is activated during metabolism of 1,2-PD, resulting in formation of bacterial microcompartments and metabolism of 1,2-PD into propionate, ATP and 1-propanol [9]. Here we show EG is metabolized into acetate and ethanol by *P. freudenreichii*, supporting additional biomass formation. Presence of EG in growth media resulted in increased abundance of proteins encoded by the *pdu* cluster, supporting evidence of its involvement in degradation of EG. Combining our results and previously reported data in *A. woodii* [18], and EG metabolism conceivably mediated by PduCDE in other microbes (discussed below), we conclude that EG is metabolized within the PDU BMC in *P. freudenreichii*.

### 4.1 Metabolic profile of *P. freudenreichii* metabolizing EG

During culturing on EG we observed biphasic growth for *P. freudenreichii* (Fig. 1A &B). After one day, a significant proportion of EG (∼9 mM) is consumed, whereas no product formation is observed and no further apparent metabolism takes place until day 4. Thereafter, more EG is metabolized and formation of ethanol and (additional) acetate is observed, together with increased biomass formation. This behaviour was previously observed for 1,2-PD utilization, where in the initial days consumption of 1,2-PD is also not associated with accumulation of 1-propanol or additional propionate [9]. Interestingly, discrepancy between 1,2-PD uptake and metabolite accumulation in initial stages is also reported for *Listeria monocytogenes* [14]. In L. *monocytogenes* this discrepancy also leads to loss of expected C_3_ compounds. It therefore is conceivable that when cells encounter EG or 1,2-PD, they accumulate these compounds in the cytosol and expression of the PDU BMC is initiated. Apparent loss of carbon in initial growth stages would be explainable by incomplete BMC formation due to BMC assembly kinetics and hence metabolism of 1,2-PD or EG by expressed diol dehydratase outside of fully assembled BMCs, leading to loss of volatile aldehydes. This hypothesis is supported by the observation that incomplete or not fully assembled BMCs in mutant strains in *Salmonella enterica* lose the ability to retain aldehydes and that BMCs function as conserving mechanism of volatiles [28]. The boiling point of acetaldehyde (20 °C) is considerably lower compared to propionaldehyde (48 °C) [50], which means the potential to exit cells as volatile gas in the used experimental conditions is higher for acetaldehyde, potentially explaining the higher observed losses of volatile intermediate on EG compared to 1,2-PD. Given the hypothesized role of BMCs for protection against aldehyde intermediates, the role of intermediate retention for EG utilization (with acetaldehyde as intermediate) seems even more relevant compared to 1,2-PD utilization in the conditions used in our experiments. Interestingly, we did not detect (detection limit 0.5 mM) acetaldehyde in culture supernatants, which implies that intermediate loss either occurs largely directly to the gas-phase due to high volatility and/or part of the acetaldehyde is lost in chemical reactions with DNA and/or proteins, resulting in formation of DNA and/or protein adducts [51]. Formation of such adducts by aldehyde interactions is supported by our findings of upregulated proteins involved in DNA repair and protein repair and stabilization. To be further supported by literature, in *S. typhimuriu*m LT2 the deletion of important DNA repair protein PolA leads to the inability to grow on ethanolamine (acetaldehyde intermediate), and 1,2-PD (propionaldehyde intermediate) and showed increased sensitivity to both propionaldehyde and acetaldehyde [46]. This requirement is likely caused by low cytoplasmic levels of toxic intermediate that are not retained or are generated outside of the BMC by alternative alcohol dehydrogenases [28], as shown by low levels of propionaldehyde in wild-type *S. enterica* [52].

Increased levels of enzymes in the cysteine biosynthesis pathway in *P. freudenreichii* observed in this study is likely a response to increased oxidative stress experienced during BMC-mediated metabolism. Cysteine likely plays a role in maintaining protein stability, as disulphide bonds formed between cysteine are important for protein folding and conformation [53]. In *Lactobacillus acidophilus* cysteine synthase is upregulated in response to oxidative stress from H_2_O_2_ [54]. Similarly, in *Staphylococcus aureus* cysteine synthase mutants were more susceptible to H_2_O_2_ [55]. Increase in oxidative stress response is also reported in other studies [9,14] and thus seems to be a general response to BMC-mediated metabolism with aldehyde intermediates.

Our results also indicate that strict anaerobic conditions enhance EG utilization (Fig. 1C). Oxygen has the potential to inactivate PDU BMC proteins, as reported for PduQ [56] and diol dehydratase encoded in *S. enterica* [57]. One potential additional role of the BMC is hypothesized to shield redox-sensitive enzymes from oxygen [58]. Daily sampling in our experiments may thus have resulted in partial Pdu enzyme inactivation, reducing the amount of EG that could be utilized.

EG grown cells showed significant increase of major shell proteins PduABJKM, diol dehydratase (PduCDE), 1-propanol dehydrogenase (PduQ), Coa-dependent propionaldehyde dehydrogenase (PduP), phosphate propanoyltransferase (PduL), corrinoid adenosyltransferase (PduO) and diol dehydratase reactivase (PduGH, annotated as DhaG) (Fig. 3). PduN was found to be more than 2-fold expressed, but did not meet the p < 0.05 treshold. AckA was detected, but no significant difference in expression was found. PduU and PduV were not detected in our proteome analysis. PduU is one of the lesser abundant Pdu-proteins [59] and is not required for correct BMC assembly [13]. The function of PduV remains unknown, although there is evidence it is implicated with spatial distribution of the BMCs within bacterial cells [60]. Theoretically it is possible BMC assembly occurs without expression of these genes, however, we find it more plausible both proteins were not detected due to low transcription of the corresponding genes, as suggested earlier for PduT in *A. woodii,* which could not be detected using mass-spectrometry but was found to be expressed using gene expression analysis [18]. Hence, a similar approach is suggested for elucidating whether or not PduU and PduV are expressed in *P. freudenreichii*. Knock-out studies could be performed to elucidate which roles these proteins have for assembly, localization and for instance substrate transport across the BMC-shell in *P. freudenreichii*.

### 4.2 The metabolism of EG by other BMC-containing organisms

EG can be produced as end-product from a variety of substrates such as xylose, arabinose, lyxose and serine. Xylose and arabinose are degradation products found after degradation of hemicellulose and thus abundant during plant-based fermentations. Xylose can be converted to xylonic acid microbiologically, with further conversions toto pyruvate, EG and glycolic acid anaerobically by the Dahms pathway (for instance by *Escherichia coli* and *Enterobacter cloacae*) and EG can be metabolized to acetate and ethanol by PduCDE containing organisms within a BMC-mediated pathway [19,61,62]. Plant-derived EG may thus be formed during food fermentations, as reported by various authors [[20], [21], [22]], and hence may act as additional carbon source for *pdu*-encoding organisms in the fermentation processes. Moreover, the presence of EG is widespread, as trace amounts are consistently detected in water sources, as well as human blood and urine [[23], [24], [25]]. This further highlights the ecophysiological significance for microorganisms with the metabolic flexibility to utilize EG in diverse biological niches including the animal microbiome.

.Evidence of EG metabolism by a BMC-mediated pathway containing a diol dehydratase as key enzyme has first been presented in *A. woodii*. In our study we have shown EG is also metabolized by *P. freudenreichii* in a BMC-mediated PDU pathway (Fig. 1, Fig. 3). Hence, it is very plausible other microorganisms containing B12-dependent BMC-PDU clusters have the capacity to metabolize EG. Alternatively, in a similar fashion B12-independent glycyl radical enzyme (GRE) microcompartments mediating 1,2-PD utilization may also support the use of EG as a substrate. As to our knowledge, no other experimental studies on the metabolism of EG and its relation to BMCs are reported in literature, except for the studies on *A. woodii* and our study. Hence the question arises whether any (or a large fraction of) BMC dependent PDU-containing organisms such as *S. typhimurium, L. monocytogenes* or *Li. reuteri* would be able to metabolize EG by a BMC-mediated PDU pathway and this topic is overlooked up to now, or whether novel types of BMC-mediated EG utilization exists.

To address this problem we identified organisms encoding *pdu* operons and/or B12-dependent diol dehydratases based on whole-genome sequencing and combine this with reported experimental evidence of these organisms utilizing EG and 1,2-PD through the same pathway as *A. woodii* and *P. freudenreichii*, which have experimentally validated BMC-mediated EG metabolism. In *Levilactobacillus brevis* the diol dehydratase encoded by *pduCDE* was purified and showed activity on 1,2-PD, glycerol and EG Ref. [63]. Likewise, a B12-dependent diol dehydratase of *Secundilactobacillus collinoides* was isolated and showed similar activity on these substrates [64]. However, these authors did not show product formation nor active metabolism/growth in these species. Growth and substrate utilization were shown for a variety of *Enterobacteriaceae* and *P. freudenreichii* on 1,2-PD and EG Ref. [65]. *Citrobacter freundii, Citrobacter intermedium* and *Klebsiella pneumoniae* were able to grow on 1,2-PD and EG. *P. freudenreichii* DSM20271 was found to grow well on 1,2-PD but barely showed any biomass differences to the basal media after 3 days incubation on EG (Fig. 1A), in line with our findings that additional biomass formation takes places after more than 3 days of incubation. The diol dehydratases encoded in these organisms formed propionaldehyde, showing the anaerobic pathway was followed. Based on their results, Toraya et al. [65] conclude that the ability to ferment 1,2-PD is closely related to the metabolism of EG. However, any evidence that this was due to PDU BMC-mediated metabolism was not presented. For *K. pneumoniae* it was confirmed the diol dehydratase responsible for converting EG to acetaldehyde was B12-dependent, as the diol dehydratase of *K. pneumoniae* was found to convert EG to acetate and ethanol in equimolar amounts only in the presence of B12. When B12 was not present as cofactor, this diol dehydratase was unable to form acetate and ethanol from EG Ref. [66], proving this enzyme indeed is B12-dependent and very likely the diol dehydratase encoded by *pduCDE* in the genome of *K. pneumoniae*. It therefore seems diol dehydratase encoding organisms often have functionality towards EG which would support a claim for a duality in functionality evolved from underground promiscuous activity of PDU proteins [67].

Thus, evidence of anaerobic EG metabolism by (B12-dependent) diol dehydratases with acetaldehyde as intermediate does exist, although most of these studies originate from before the discovery of BMCs*. A. woodii, P. freudenreichii, C. freundii, Se. collinoides, Le. brevis* and *K. pneumoniae* all have in common that they contain the PDU BMC cluster (based on information from BMC-caller [9,18,68]. Based on the experimental validated activity on 1,2-PD and EG, reports of propionaldehyde/acetaldehyde intermediate formation and genomic information of these organism it is thus very likely EG is metabolized by the PDU BMC-mediated pathway. Interestingly, *C. freundii* and *K. pneumoniae* also encode EUT BMCs, which also have acetaldehyde as intermediate and acetate and ethanol as end-product. Although the signature enzyme ethanolamine ammonia-lyase likely does not display activity on EG, it cannot be excluded (part of the) EUT BMCs can be involved in EG metabolism, as the EUT shell and rest of the pathway may be better adapted for metabolism of EG due to the similarity in intermediates. The fact that multiple reports of PDU BMC-mediated glycerol utilization exist [[69], [70], [71]] suggests more broad substrate specificity for the PDU microcompartment, showing also activity on EG and/or glycerol in addition to 1,2-PD conversion.

The enhanced stress resistance in *P. freudenreichii* under BMC-inducing conditions (Fig. 6) as demonstrated in this study, also highlights the evolutionary and ecological significance of the presumably widespread BMC-mediated metabolism and its broad substrate specificity. Besides extending the metabolic capacity, the elevated stress response can also increase the robustness of the bacterium in adverse environments [72]. For *P. freudenreichii*, the elevated stress resistance against low pH and bile salts may support its growth and survival in the gastrointestinal tract, further establishing its potential as a probiotic species [73].

## 5 Conclusions

*P. freudenreichii* is able to metabolize EG to acetate and ethanol whilst benefiting energetically. Addition of EG to the growth medium results in significant higher expression of PDU proteins and DNA repair proteins. BMC structures were observed using TEM and the upregulated stress proteins in EG-grown cells provide protection against acid and bile salt-induced stresses. As in *A. woodii*, EG is metabolized in a PDU BMC-dependent pathway. Evidence of other BMC-containing organisms metabolizing EG with the same diol dehydratase responsible for 1,2-PD metabolism implies the PDU BMC encodes for EG metabolism in multiple organisms. Experimental studies on EG metabolism in PDU BMC-containing organisms and the effect of EG on PDU BMC expression need to be performed to determine whether this phenomenon is widespread. Reports of PDU BMC-mediated glycerol utilization and our results indicate the *pdu* cluster encodes for proteins with a more broad substrate specificity with activity on EG and/or glycerol, in addition to 1,2-PD. The importance of the PDU BMC in pathogenic *pdu*-encoding organisms and possible impact on food safety also requires attention. The role in beneficial microbes including *P. freudenreichii*, during food fermentations and plant-derived EG metabolism in the human intestine, remains to be elucidated.

## Dataset citation

Dank, Alexander et al. (2021) data from: “Bacterial Microcompartment-Dependent 1, 2-Propanediol Utilization of *Propionibacterium freudenreichii*.” Frontiers in microbiology (2021): 1127. Available at PRIDE repository under identifier PXD024700 at: https://www.ebi.ac.uk/pride/archive/projects/PXD024700.

## Data availability statement

The mass spectrometry proteomics data have been deposited to the ProteomeXchange Consortium via the PRIDE partner repository with the dataset identifier PXD037369. Other research data from this study can be found in the additional files supplied, or will be made available on request.

## CRediT authorship contribution statement

**Alexander Dank:** Writing – review & editing, Writing – original draft, Visualization, Investigation. **Yue Liu:** Writing – review & editing, Visualization, Supervision, Formal analysis. **Xin Wen:** Methodology, Investigation. **Fan Lin:** Investigation. **Anne Wiersma:** Formal analysis. **Sjef Boeren:** Resources, Methodology, Formal analysis. **Eddy J. Smid:** Writing – review & editing, Supervision, Project administration, Conceptualization. **Richard A. Notebaart:** Conceptualization. **Tjakko Abee:** Writing – review & editing, Supervision, Conceptualization.

## Declaration of competing interest

The authors declare the following financial interests/personal relationships which may be considered as potential competing interests: Alexander Dank reports financial support was provided by Arla Foods amba.
