# Supplementary material for: Ethylene glycol is metabolized to ethanol and acetate and induces expression of bacterial microcompartments in Propionibacterium freudenreichii
Source: Heliyon. 2024 Jun 22;10(13):e33444. doi: 10.1016/j.heliyon.2024.e33444 (PMC11255663; doi:10.1016/j.heliyon.2024.e33444)
Supplement: Multimedia component 1 [file mmc1.pdf]

Supplementary table 1: Upregulated proteins of *Propionibacterium freudenreichii* grown in Ethylene glycol media compared to *P. freudenreichii* grown in Lactate media. Proteins are upregulated at least 2-fold with a FDR of <0.05.

Protein ID (Uniparc)	Protein Name	Log2 difference	P-value
UPI00005B73B3	1-propanol dehydrogenase PduQ	2.500313936	0.001213924
UPI00005B73C1	Ribose-5-phosphate isomerase 3	1.289510594	0.00032388
UPI00005B73C4	Transaldolase	1.556123654	0.000204968
UPI0001BFF0E9	CoA-dependent propionaldehyde dehydrogenase	7.853431389	0.000268496
UPI0001D5C614	Carbon dioxide concentrating mechanism/carboxysome shell protein	6.962152614	2.02E-07
UPI0001D5C615	Propanediol utilization protein PduB	10.50220643	1.31E-05
UPI0001D5C616	Glycerol dehydratase large subunit	7.289867086	2.22E-06
UPI0001D5C682	Transcription regulator	1.188709449	0.002852355
UPI0001D5C713	DSBA oxidoreductase	1.353366086	0.00073759
UPI0001D5C785	Starvation-inducible DNA-binding protein	1.138147042	0.008889069
UPI0001D5C787	Dehydratase small subunit	7.479831063	1.13E-06
UPI0001D5C78A	Propanediol utilization protein PduK	7.273053255	0.000130894
UPI0001D5C79E	Deoxyguanosinetriphosphate triphosphohydrolase (DGTPase)	1.216727572	0.024519762
UPI0001D5C8A9	50S ribosomal protein L27	1.134550263	0.005145389
UPI0001D5C8AE	Gamma-glutamyl phosphate reductase	1.392322915	0.010601038
UPI0001D5CB9A	AMMECR1 domain protein	1.094766048	0.000427009
UPI0001D5CBF9	Amino acid permease-associated region	1.061877067	0.009965823
UPI0001D5CBFC	Ppx/GppA phosphatase family	1.021743312	0.000331982
UPI0001D5CBFD	Uncharacterized protein	1.384351605	0.002913348
UPI0001D5CC44	Holliday junction ATP-dependent DNA helicase RuvB	1.142627175	0.001298153
UPI0001D5CD00	YhgE/Pip domain protein	1.048416604	0.043345383
UPI0001D5CD56	RNA polymerase principal sigma factor HrdD	2.354257375	0.002764804
UPI0001D5CD6D	Inositol 2-dehydrogenase IdhA	1.305290061	0.000939254
UPI0001D5CD6F	IolC (Myo-inositol catabolism iolC protein)	1.043244255	0.002607033
UPI0001D5CD9F	Lactaldehyde dehydrogenase	1.20522654	0.001420693
UPI0001D5CDB9	Branched-chain amino acid aminotransferase	1.315311091	0.000911636
UPI0001D5CED6	Pyridoxal 5'-phosphate synthase subunit PdxT	4.413928975	1.03E-05
UPI0001D5CED7	Pyridoxal 5'-phosphate synthase subunit PdxS	4.021845789	2.53E-06
UPI0001D5CF17	UvrABC system protein B	1.355793331	0.000869836
UPI0001D5CF91	Sulfate adenylyltransferase subunit 1	3.762431038	0.000145485
UPI0001D5D0FF	Proteasome endopeptidase complex	1.280446311	0.000980636
UPI000323070B	"Haloacid dehalogenase superfamily enzyme subfamily IA"	1.07920148	0.000638852

UPI000323AE06	Diaminopimelate epimerase	1.086419326	0.002917495
UPI000324BD76	RNA methyltransferase	1.422096132	0.002849384
UPI0003267E56	Propanediol dehydratase medium subunit PduD	9.998640002	2.82E-06
UPI0004A035A5	"ABC-type transport systems periplasmic component"	2.633060516	0.000798449
UPI0004A0667C	ATP-dependent DNA helicase	1.049788364	0.000920272
UPI0004A0D7A3	Phosphate propanoyltransferase PduL	4.103820871	3.85E-05
UPI000541B9EB	Uncharacterized protein	3.738367153	3.10E-06
UPI000541CF70	Formamidopyrimidine-DNA glycosylase (DNA-formamidopyrimidine glycosylase)	2.241622026	0.000557497
UPI000541F2C5	DNA repair protein RadA	1.382375277	6.12E-05
UPI000541F3EC	IolE (Myo-inositol catabolism IolAEprotein) (Inosose dehydratase) (2-keto-myo-inositol dehydratase)	1.356213096	0.008578612
UPI0005420BD8	Propanediol utilization diol dehydratase reactivation protein PduG	4.137953307	0.000133255
UPI00054232DF	UvrD/REP helicase / ATP-dependent DNA helicase	1.478166171	0.002283827
UPI00054244B7	Oxidoreductase	1.602232856	4.78E-05
UPI000542557F	Corrinoid adenosyltransferase	5.844251444	1.97E-06
UPI0005428C14	"ABC transporter ATP binding protein"	3.216464126	3.72E-05
UPI0005428DED	Carbon starvation protein	4.182163271	0.002073179
UPI0005429954	ABC transporter permease	1.05540107	0.005703224
UPI000542A8A2	Propanediol utilization protein PduJ	7.038405693	2.31E-06
UPI000542B500	Protein-tyrosine phosphatase	1.250518388	0.042556379
UPI000542D616	NAD-dependent protein deacetylase	1.122553433	0.003761038
UPI0005431885	IolG2 (Myo-inositol catabolism IolG2 protein) (Inositol 2-dehydrogenase)	1.14945852	4.02E-05
UPI0005435146	IolT3 (Myo-inositol transporter iolT3)	1.310278664	0.000246774
UPI00054358A2	ATP binding protein of ABC transporter	1.49628893	2.75E-05
UPI00054367DC	Probable primosomal protein N'	1.123324323	0.007753202
UPI00054370E7	Propanediol utilization microcompartment protein PduM	5.683171521	0.005637349
UPI00054373F7	Sulfite reductase [ferredoxin]	3.203908666	0.000271888
UPI0005438BBC	Acetyltransferase family protein	1.614952527	0.004311872
UPI000543AC22	Sulfate adenylyltransferase subunit 2	1.590304667	0.004769763
UPI000543D7D1	Drug exporters of the RND superfamily	1.571944306	3.78E-05
UPI000543DF9A	Leucyl/phenylalanyl-tRNA-protein transferase	1.407034753	0.003348892
UPI0005440BDD	MEMO1 family protein PFR_JS17-1_19	1.03727092	0.038627692
UPI0005441692	Glutamate decarboxylase	1.059917108	0.041292093
UPI00054435B7	Two component sensor kinase	1.038407189	0.00399208
UPI0005443D9B	Cysteine synthase	2.958105388	0.000940774
UPI000544453F	Catalase	1.394799264	4.58E-05
UPI0005445352	Hypothetical membrane protein	3.100424721	1.50E-05

UPI0005D83E16	Large conductance mechanosensitive channel<a0>	1.017169722	0.001029655
UPI0005DA3579	1-deoxy-D-xylulose-5-phosphate synthase<a0>	1.293937926	0.048430703

Supplementary table 2: Proteins upregulated in *Propionibacterium freudenreichii* grown in either 1,2-Propanediol containing media or ethylene glycol containing media compared to lactate containing media. Proteins are upregulated at least 2-fold.

Protein ID (Uniparc)	Protein Name
**Pdu Proteins**	
UPI0001D5C614	Carbon dioxide concentrating mechanism/carboxysome shell protein PduA
UPI0001D5C615	Propanediol utilization protein PduB
UPI0001D5C616	Glycerol dehydratase large subunit PduC
UPI0003267E56	Propanediol dehydratase medium subunit PduD
UPI0001D5C787	Dehydratase small subunit PduE
UPI0005420BD8	diol dehydratase reactivase PduG
UPI000542A8A2	Propanediol utilization protein PduJ
UPI0001D5C78A	Propanediol utilization protein PduK
UPI0004A0D7A3	Phosphate propanoyltransferase PduL
UPI00054370E7	Propanediol utilization microcompartment protein PduM
UPI0001D5C78E	Propanediol utilization protein PduN
UPI000542557F	cob(I)yrinic acid a,c-diamide adenosyltransferase PduO
UPI0001BFF0E9	CoA-dependent propionaldehyde dehydrogenase pduP
UPI00005B73B3	1-propanol dehydrogenase PduQ
**DNA and RNA repair proteins/regulatory proteins**	
UPI00054232DF	UvrD/REP helicase / ATP-dependent DNA helicase
UPI0001D5CF17	UvrABC system protein B
UPI000541CF70	Formamidopyrimidine-DNA glycosylase (DNA-formamidopyrimidine glycosylase)
UPI000541F2C5	DNA repair protein RadA
UPI000541B9EB	RtcB family protein
UPI0004A0667C	ATP-dependent DNA helicase
UPI00054367DC	Probable primosomal protein N'
UPI000542D616	NAD-dependent protein deacetylase; Sir2-like transcriptional regulator
UPI0005428DED	Carbon starvation protein
UPI00054435B7	Two component system sensor kinase
**Vitamin B6 synthesis**	
UPI0001D5CED6	Pyridoxal 5'-phosphate synthase subunit PdxT
UPI0001D5CED7	Pyridoxal 5'-phosphate synthase subunit PdxS
**Sulfur metabolism/Protein synthesis/Protein recycling**	
UPI0005443D9B	Cysteine synthase
UPI000543AC22	Sulfate adenylyltransferase subunit 2
UPI00054373F7	Sulfite reductase [ferredoxin]

UPI0004A035A5	ABC-type transport systems, periplasmic component; ABC transporter methionine
UPI0001D5C713	DSBA oxidoreductase
UPI0001D5CBF9	Amino acid permease-associated region
UPI0001D5C8AE	Gamma-glutamyl phosphate reductase
UPI0001D5CBA5	50S ribosomal protein L33
UPI000543DF9A	leucyl/phenylalanyl-tRNA–protein transferase
UPI000323AE06	Diaminopimelate epimerase
**Putative transporters**	
UPI0005428C14	ABC transporter, ATP binding protein
UPI000543D7D1	Membrane protein YdfJ
**Various/unknown function**	
UPI00054244B7	Oxidoreductase
UPI0005438BBC	Acetyltransferase family protein
UPI0001D5CB9A	AMMECR1 domain protein
UPI0001D5CBFC	Ppx/GppA phosphatase family
UPI0005440BDD	MEMO1 family protein PFR_JS17-1_19
UPI000542DA5A	Methyltransferase type 11
UPI0001D5CD9F	Lactaldehyde dehydrogenase
UPI0001D5CFDE	PF10708 family protein
